# Effectiveness of single-dose methotrexate in the treatment of ectopic pregnancy: a retrospective study

**DOI:** 10.61622/rbgo/2026rbgo28

**Published:** 2026-07-17

**Authors:** Letícia Pereira Mendonça, Eduardo Cunha da Fonseca

**Affiliations:** 1 Hospital Mater Dei Belo Horizonte MG Brasil Hospital Mater Dei, Belo Horizonte, MG, Brasil

**Keywords:** Ectopic pregnancy, Methotrexate, Effectiveness, Pelvic pain, Treatment outcome, Treatment failure

## Abstract

**Objective:**

Evaluate the effectiveness of Methotrexate in clinical treatment of ectopic pregnancy and analyze the influence of clinical and laboratory variables on the treatment outcome.

**Methods:**

It was a retrospective study of 95 patients undergoing clinical treatment with Methotrexate at a private tertiary hospital in the metropolitan region of Belo Horizonte. Variables such as age, presence of pelvic pain, previous ectopic pregnancy, initial beta-hCG level and largest adnexal mass measurement were analyzed. The theoretical framework followed a data search in PubMed, selecting 17 articles in total.

**Results:**

The clinical success rate with a dose of Methotrexate was 71,6%. Among the variables evaluated, only the initial beta-hCG value showed statistical significance with the outcome, therefore, higher the beta-hCG, lower the chance of treatment effectiveness, with a p-value of 0.002. The other variables analyzed did not show any significant association.

**Conclusion:**

Methotrexate has been shown to be effective in most cases of ectopic pregnancy, with the initial beta-hCG value standing out as a predictor of treatment failure. The findings reinforce the importance of adequate patient selection and laboratory monitoring in clinical follow-up.

## Introduction

Ectopic pregnancy (EP) is a significant cause of maternal morbidity and mortality in the first trimester of pregnancy. Its incidence has increased in recent decades, largely due to improved diagnostic accuracy through transvaginal ultrasonography and the measurement of serum beta-hCG levels. It is a common cause of abdominal pain in gynecology and has an incidence of 2 to 3% in all pregnancies. Early diagnosis has made conservative treatments more viable, particularly medical management with methotrexate (MTX), which aims to avoid surgical intervention and preserve fertility.^(1-4)^

MTX is a folic acid antagonist that inhibits DNA synthesis and cell division. In the context of EP, it promotes trophoblastic tissue regression. The single-dose protocol is commonly used due to its simplicity and favorable safety profile. However, treatment success is not guaranteed and may be influenced by clinical and laboratory factors, including initial beta-hCG levels, size of the adnexal mass, presence of fetal cardiac activity, and patient symptoms.^(2,3,5,6)^

Although several studies have evaluated the efficacy of MTX, most were conducted in public hospitals, where patient profiles and health service dynamics differ from those in private institutions. Thus, it is important to understand the effectiveness of MTX in different healthcare contexts and identify variables associated with treatment failure.

The National Institute for Health and Care Excellence (NICE) in England recommends the use of methotrexate as first-line therapy for women who can undergo follow-up and meet the following criteria: 1. They have no significant pain. 2. An uninterrupted ectopic pregnancy with a mass smaller than 35 mm without a visible heartbeat. 3. A beta-hCG level below 1500 IU/L without an intrauterine pregnancy confirmed by ultrasound.^(5,7)^

The serum Beta-HCG levels are measured on days 4 and 7 after the medication. If there is a reduction of more than 15% between days 4 and 7, the levels should be measured weekly until they are below 15 mIU/L. If there is no 15% reduction, the administration of a second dose of Methotrexate may be considered.^(8)^

Overall, the success rate of Methotrexate for ectopic pregnancy ranges between 65–95%, with 3–27% requiring a second dose. Some factors are considered predictors of success, including an initial Beta-HCG level below 1000 mIU/L, absence of a gestational sac on ultrasound, minimal increase of Beta-HCG prior to medication administration, and a decline in Beta-HCG between days 1 and 4.^(2,3,5)^

It is important to emphasize to patients undergoing medical treatment that Methotrexate does not affect future reproductive function. However, it is recommended that women who have used Methotrexate wait at least 3 months before attempting conception again.^(5,9)^ On the other hand, failure of clinical treatment is associated with the need for emergency surgical intervention and possible impacts on fertility. Therefore, early identification of prognostic factors, such as elevated beta-hCG levels, is essential to prevent serious complications.^(9,10)^

This study aimed to evaluate the effectiveness of single-dose MTX in the treatment of ectopic pregnancy at a private tertiary hospital and to analyze clinical and laboratory factors associated with therapeutic success or failure.

## Methods

This was a retrospective, observational study conducted at a private tertiary hospital in the metropolitan region of Belo Horizonte, Brazil, from 2019 to 2023.

The inclusion criteria were women aged ≥18 years, diagnosis of ectopic pregnancy confirmed by clinical criteria and transvaginal ultrasound, clinical stability and eligibility for medical treatment with Methotrexate. The exclusion criteria were contraindications to Methotrexate, such as anemia, leucopenia, thrombocytopenia, impaired renal and hepatic function, hypersensitivity, hemodynamic instability, signs of rupture, initial serum beta-hCG >5,000 mIU/mL, presence of fetal cardiac activity on ultrasound, adnexal mass > 4 cm, incomplete medical records or loss to follow-up.

Patients received a single intramuscular dose of methotrexate (50 mg/m^2^ of body surface area). Serum beta-hCG levels were monitored immediately before MTX administration, defined as day 1, and subsequently on days 4, and 7 post-treatment. A ≥15% decrease in beta-hCG from day 4 to day 7 was considered indicative of treatment response. Patients not meeting this criterion were offered a second MTX dose. Expectant management is not part of the routine practice at this institution; methotrexate is the standard therapeutic approach for all eligible patients.

The variables analyzed included: age, parity, presence of pelvic pain, history of previous ectopic pregnancy, initial beta-hCG level, and maximum diameter of the adnexal mass. Patients were categorized into two groups based on treatment outcome: success (resolution without surgery) and failure (requiring surgical intervention).

The theoretical framework was obtained through a systematic review in the PubMed database using the keywords "ectopic pregnancy," "methotrexate," and "effectiveness," covering the years 2019 to 2024, which resulted in 242 studies. Paid texts were excluded, leaving 144 results. After excluding articles based on titles that addressed unrelated topics, 28 articles remained. Finally, 13 articles were selected after full-text reading.

Although the time frame of the literature review was limited to 2019–2024, three articles from previous years were intentionally and carefully included due to their ongoing clinical and conceptual relevance in the current context. The guideline of the Royal College of Obstetricians and Gynaecologists remains an international reference for the diagnostic and therapeutic management of ectopic pregnancy and is widely cited by contemporary studies. The work of Stovall et al. (1991)^(8)^ describes the original single-dose Methotrexate protocol, which serves as the basis for virtually all subsequent clinical studies on the subject. Bourne et al. (2013)^(7)^ present a well-founded critique of the NICE guidelines, addressing practical and ethical implications regarding treatment accessibility, making it relevant for discussions on public health and patient safety. Therefore, their inclusion was justified by historical, clinical, and methodological relevance, as well as the lack of recent publications that fully replace their content.

Descriptive statistics were presented as means and standard deviations or medians and interquartile ranges, depending on distribution. Comparisons between groups were made using Student's t-test, Mann–Whitney U test, chi-square test, or Fisher's exact test, as appropriate. Multivariate logistic regression was performed to identify independent predictors of treatment failure. Statistical significance was set at p<0.05.

The research was approved by the institution's Research Ethics Committee under protocol number *Certificado de Apresentação para Apreciação Ética*: 78267124.5.0000.5128, Process nº 6.798.288 of May 01, 2024.

## Results

**Figure 1:**
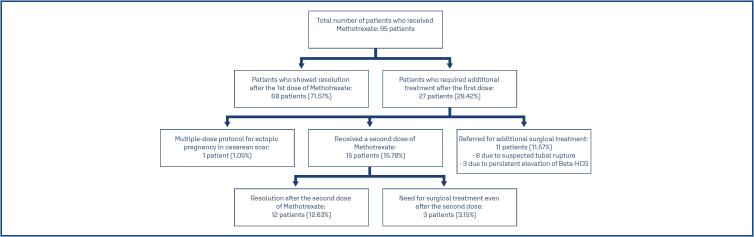
Flow of the patients observed in the study according to treatment and success rate. 71.6% of the patients achieved clinical success with a single dose of Methotrexate, while 12.6% showed resolution with treatment using two doses. 1% required treatment with multiple doses, and in total, 14 patients underwent additional surgical treatment for resolution

**Figure 2:**
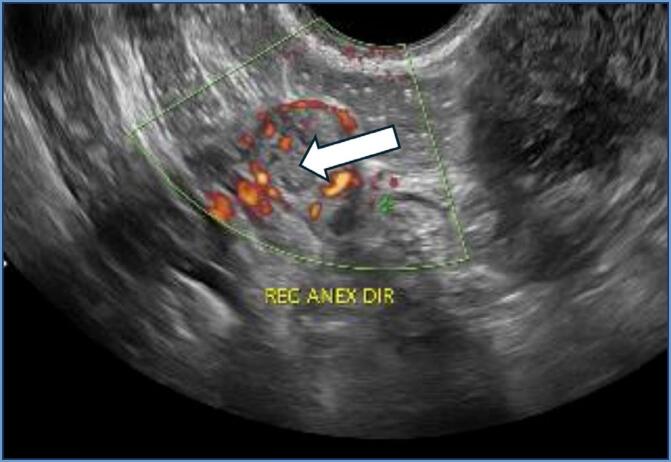
Transvaginal ultrasound from the study cohort, showing a right adnexal mass with peripheral vascularization (arrow), consisent with the classical tubal ring sign of ectopic pregnancy

A total of 95 patients with a confirmed diagnosis of ectopic pregnancy were treated with methotrexate between 2019 and 2023. The mean age of the participants was 30.8 years (±5.4), and the median initial beta-hCG level was 1,436 mIU/mL (interquartile range: 798–2,507). Most patients (83.2%) reported pelvic pain at the time of diagnosis, and 18.9% had a previous history of ectopic pregnancy. The mean diameter of the adnexal mass was 2.9 cm (±0.9), and no cases presented with fetal cardiac activity. (Figure 1). An illustrative example of an adnexal mass on transvaginal ultrasound from our patient cohort is shown in figure 2.

The success rate of clinical treatment with a single dose of methotrexate was 71.6% (68/95 patients). When patients who required a second dose were included, the total success rate raised to 84.2% (80/95). Fifteen patients (15.8%) did not respond to medical treatment and required surgical intervention (laparoscopy or laparotomy) due to increased beta-hCG levels, persistence of the adnexal mass, or clinical worsening. The variables age, initial beta-hCG level, and adnexal mass diameter were compared between the groups with and without treatment failure. The mean age was 33.9 ± 4.9 years in the clinical success group and 35.6 ± 4.9 years in the group that required additional treatment, with no statistically significant difference (p = 0.139). The initial beta-hCG level was significantly higher in the failure group (1993.0 ± 1289.8 IU/L) compared to the success group (1059.9 ± 1113.8 IU/L), with a statistically significant difference (p = 0.002) (Table 1). The largest measurement of the adnexal mass was 20.8 ± 7.1 mm in the success group and 18.4 ± 7.9 mm in the failure group, with no statistical significance (p = 0.167).

**Table 1:** Distribution of patients across three initial beta-hCG level ranges based on treatment outcome. There is a trend toward a higher failure rate among patients with levels above 1000 IU/L, particularly those above 3000 IU/L. The difference was statistically significant (p = 0.002)

Beta-hCG	Clinical success	Failure	Total
< 1000	43	7	50
1000–3000	19	13	32
> 3000	6	7	13
Total	68	27	95

A statistically significant association was found between initial beta-hCG level and treatment failure (p = 0.002). Patients in the failure group had higher mean beta-hCG levels (2,580 mIU/mL) compared to the success group (1,268 mIU/mL). Other variables such as age, parity, pelvic pain, previous ectopic pregnancy, and adnexal mass diameter showed no significant differences between the groups (Table 2).

**Table 2:** Variables analyzed with treatment success and failure outcomes, along with p-values

Variables	Mean ± SD	Mean ± SD	p-value
Age (Years)	33.9 ± 4.9	35.6 ± 4.9	0.139
Initial B-hCG (IU/L)	1059.9 ± 1113.8	1993.0 ± 1289.8	0.002
Largest mass measurement (mm)	20.8 ± 7.1	18.4 ± 7.9	0.167

SD - standard deviation

A logistic regression analysis was performed to evaluate predictors of treatment success. In the univariate model, serum β-hCG levels between 2001–5000 mIU/mL were significantly associated with lower odds of success (OR 0.23; 95% CI 0.09–0.61; p = 0.003). In the multivariate analysis, this variable remained an independent predictor of treatment failure (OR 0.21; 95% CI 0.07–0.62; p = 0.005). Other variables, including age, parity, previous ectopic pregnancy, adnexal mass diameter, and pelvic pain, were not significantly associated with outcomes (Table 3).

**Table 3:** Logistic regression analysis of factors associated with methotrexate treatment success in ectopic pregnancy

Variables	Univariate OR	95% CI	p-value	Multivariate OR	95% CI	p-value
Age (Years)	0.93	0.84–1.02	0.138	0.94	0.84–1.05	0.231
Parity	1.00	0.71–1.41	0.996	1.10	0.73–1.67	0.645
Previous ectopic pregnancy	0.77	0.21–2.79	0.687	1.63	0.32–8.43	0.559
B-hCG 200–105000 mIU/mL	0.23	0.09–0.61	0.003	0.21	0.07–0.62	0.005
Mass diameter (mm)	1.05	0.98–1.12	0.146	1.07	0.99–1.15	0.078
Pelvic pain	1.02	0.40–2.63	0.964	0.63	0.21–1.87	0.402

OR – Odds Ratio; CI - confidence interval

**Note**: For regression analysis, beta-hCG values were categorized into two ranges (≤2000 and 2001–5000 mIU/mL), as no patients in this cohort presented levels above 5000 mIU/mL.

In multivariate logistic regression, only the initial beta-hCG level remained an independent predictor of treatment failure (p < 0.01), reinforcing its prognostic importance.

## Discussion

This study evaluated the effectiveness of single-dose methotrexate in the treatment of ectopic pregnancy at a private tertiary hospital, finding a clinical success rate of 71.6% with one dose, increasing to 84.2% when including patients who required a second dose. These results are consistent with previous studies reporting success rates ranging from 68% to 95%, depending on inclusion criteria and treatment protocols.^(1,3,4,9,10)^

In 2017, a retrospective study was conducted at the same hospital unit, including 84 patients diagnosed with ectopic pregnancy who received medical treatment with Methotrexate. In 68 patients, medical treatment—considering one or two doses—was successful (80.95%), a result that supports the data found in the current study, where an 84% success rate was observed for medical treatment considering one and two doses.^(11)^

Among the variables analyzed in the current study, the initial beta-hCG level was the only factor significantly associated with treatment outcome. Patients who required additional treatment, either with a second dose or via videolaparoscopy, had significantly higher initial levels (1993.0 ± 1289.8 IU/L) compared to those who achieved clinical resolution (1059.9 ± 1113.8 IU/L), with p = 0.002. This finding reinforces what is widely described in the literature: higher beta-hCG levels at the time of diagnosis are associated with a greater risk of medical treatment failure, possibly reflecting greater trophoblastic activity and a larger volume of gestational tissue. Most references cite a cutoff value of 5,000 mIU/mL; however, one study suggests that patients with beta-hCG > 3,000 mIU/mL may require a second dose, as it demonstrated an 82% success rate in patients receiving two doses, compared to 67% in those treated with a single Methotrexate dose.^(1,3,4,6,10,12-14)^

Another study conducted in 2024, focused on cases with higher beta-hCG levels (above 5,000 mIU/mL), concluded that Methotrexate can still be effective in such cases. However, the need for strict monitoring becomes even greater due to the higher risk of treatment failure.^(15)^

Although slightly older, patients who required additional treatment did not show a statistically significant difference in age (p = 0.139). Similarly, the largest measurement of the adnexal mass did not differ significantly between the groups (p = 0.167), suggesting that, in this sample, the size of the lesion observed on ultrasound was not predictive of treatment failure. Nevertheless, the ultrasound aspect remains an important diagnostic feature. In our cohort, adnexal masses typically appeared as heterogeneous structures adjacent to the ovary, sometimes presenting the classical hyperechoic "tubal ring sign," widely described in the literature as a hallmark of tubal ectopic pregnancy. This finding, illustrated in Figure 2, highlights the relevance of ultrasound evaluation in guiding clinical decision-making and supports its role as a complementary tool to biochemical markers. The presence of pelvic pain, which is often considered an exclusion criterion for medical treatment in some guidelines, was not associated with therapeutic failure (p = 0.402). The same was observed for previous ectopic pregnancy (p = 0.559). These findings indicate that such factors, in isolation, should not be considered absolute contraindications to the use of Methotrexate, provided the patient is hemodynamically stable and meets the appropriate clinical criteria. Other factors have already been described in the literature as being of lesser utility for predicting success.^(3,4,9,10,12,16,17)^

These results are consistent with previous studies that identify beta-hCG as the main predictive marker of therapeutic success. However, the study has limitations, including its retrospective design, reliance on medical records, and the use of a fixed methotrexate dose rather than dosing based on body surface area in some cases, which may have led to underdosing in heavier patients and influenced outcomes.

Despite these limitations, the findings contribute to clinical practice by reinforcing that beta-hCG levels remain the strongest predictor of medical treatment success and support the use of methotrexate as a safe and effective option in carefully selected patients.

## Conclusion

Single-dose methotrexate is an effective and safe treatment for ectopic pregnancy in most cases, with an overall success rate of 71.6% with one dose, increasing to 84.2% when a second dose is administered. Among the variables analyzed, only the initial beta-hCG level was significantly associated with treatment failure, highlighting its role as a key prognostic factor. Other clinical variables such as age, parity, adnexal mass size, pelvic pain, and previous ectopic pregnancy did not influence treatment outcomes, suggesting that these factors alone should not contraindicate medical management in clinically stable patients. Future prospective studies with larger sample sizes and standardized dosing protocols are recommended to validate these findings and further refine patient selection criteria for medical treatment.

## Data Availability

The research data are described in the article presented.
